# Mothers' and fathers' joint profiles for testosterone and oxytocin in a small‐scale fishing‐farming community: Variation based on marital conflict and paternal contributions

**DOI:** 10.1002/brb3.1367

**Published:** 2019-08-05

**Authors:** Lee T. Gettler, Mallika S. Sarma, Sheina Lew‐Levy, Angela Bond, Benjamin C. Trumble, Adam H. Boyette

**Affiliations:** ^1^ Department of Anthropology University of Notre Dame Notre Dame Indiana; ^2^ Eck Institute for Global Health University of Notre Dame Notre Dame Indiana; ^3^ William J. Shaw Center for Children and Families University of Notre Dame South Bend Indiana; ^4^ Department of Psychology University of Cambridge Cambridge UK; ^5^ School of Human Evolution and Social Change Arizona State University Tempe Arizona; ^6^ Center for Evolution and Medicine Arizona State University Tempe Arizona; ^7^ Thompson Writing Program Duke University Durham North Carolina; ^8^ Max Planck Institute for Evolutionary Anthropology Department of Human Behavior, Ecology and Culture Leipzig Germany

**Keywords:** androgens, cross‐cultural comparison, endocrine system, family health, hormones, interparental conflict, parenting

## Abstract

**Introduction:**

Testosterone and oxytocin are psychobiological mechanisms that interrelate with relationship quality between parents and the quantity and quality of parenting behaviors, thereby affecting child outcomes. Their joint production based on family dynamics has rarely been tested, particularly cross‐culturally.

**Methods:**

We explored family function and salivary testosterone and oxytocin in mothers and fathers in a small‐scale, fishing‐farming society in Republic of the Congo. Fathers ranked one another in three domains of family life pertaining to the local cultural model of fatherhood.

**Results:**

Fathers who were viewed as better providers had relatively lower oxytocin and higher testosterone than men seen as poorer providers, who had lower testosterone and higher oxytocin. Fathers also had higher testosterone and lower oxytocin in marriages with more conflict, while those who had less marital conflict had reduced testosterone and higher oxytocin. In contrast, mothers in conflicted marriages showed the opposite profiles of relatively lower testosterone and higher oxytocin. Mothers had higher oxytocin and lower testosterone if fathers were uninvolved as direct caregivers, while mothers showed an opposing pattern for the two hormones if fathers were seen as involved with direct care.

**Conclusions:**

These results shed new light on parents' dual oxytocin and testosterone profiles in a small‐scale society setting and highlight the flexibility of human parental psychobiology when fathers' roles and functions within families differ across cultures.

## Résumé

**Introduction:**

La testostérone et l'ocytocine sont des mécanismes psychobiologiques qui sont interdépendants de la qualité de la relation entre les parents, ainsi que de la quantité et la qualité des comportements parentaux, et ainsi affectent les résultats de l'enfant. Leur production conjointe basée sur les dynamiques familiales a rarement été testée, en particulier de manière transculturelle.

**Méthodeis:**

Nous avons investigué la fonction familiale, la testostérone et l'ocytocine chez les mères et les pères d'une micro société de pêcheurs‐agriculteurs en République du Congo. Les pères se sont eux‐mêmes classés dans trois domaines de la vie familiale relatif au modèle culturel local de la paternité.

**Résultatis:**

Les pères qui étaient considérés comme de meilleurs pourvoyeurs avaient relativement moins d'ocytocine et plus de testostérone que les hommes considérés comme des moins bons pourvoyeurs, ces derniers ayant des taux de testostérone plus bas et des taux d'ocytocine plus haut. Les pères présentaient également des taux plus élevés de testostérones et plus bas d'ocytocine dans les mariages plus conflictuels, alors que ceux engagés dans des mariages moins conflictuels présentaient une testostérone réduite et une ocytocine plus élevée. En revanche, les mères dans les mariages en conflit ont montré les profils opposés de testostérone relativement faible et d'ocytocine plus élevée. Les mères avaient des taux d'ocytocine et de testostérone plus élevés si les pères n'étaient pas impliqués directement dans les soins, tandis que les mères présentaient un schéma opposé pour les deux hormones si les pères étaient considérés comme impliqués dans les soins directs.

**Conclusions:**

Ces résultats ont jeté un nouvel éclairage sur les profils doubles de l'ocytocine et de la testostérone chez les parents, dans un nouveau contexte de micro société et ont mis en évidence la flexibilité de la psychobiologie parentale humaine lorsque les rôles et les fonctions des pères au sein des familles varient d'une culture à l'autre.

## 1 INTRODUCTION

Testosterone (T) and oxytocin (OT) are neuroendocrine mechanisms that influence human social behavior, cognition, and emotion, including in the context of invested social relationships (Feldman, 2012; Gettler, 2014; Rilling, 2013; Storey & Ziegler, 2016; Trumble, Jaeggi, & Gurven, 2015; van Anders, Goldey, & Kuo, 2011). Individuals with elevated T tend to be more oriented toward competition and status seeking, while those with lower T are often more nurturant and prosocial, particularly in the context of parenting and partnering (Gettler, 2014; Gray, McHale, & Carre, 2017; van Anders, Goldey, & Kuo, 2011; van Anders, 2013). Specifically, research in multiple societies has shown that men and women often have lower T when they have young children that require intensive childcare (Alvarado et al., 2015; Barrett et al., 2013; Gray, Kahlenberg, Barrett, Lipson, & Ellison, 2002; Kuzawa, Gettler, Huang, & McDade, 2010). In some cultural settings, fathers also have reduced T when they engage in more nurturant, direct caregiving (Alvergne, Faurie, & Raymond, 2009; Edelstein et al., 2017; Gettler, McDade, Agustin, Feranil, & Kuzawa, 2015; Kuo et al., 2018; Mascaro, Hackett, & Rilling, 2013; Weisman, Zagoory‐Sharon, & Feldman, 2014). U.S. men and women with lower T also report greater commitment and satisfaction in their romantic relationships, as do their partners (Edelstein, van Anders, Chopik, Goldey, & Wardecker, 2014; Saxbe, Edelstein, et al., 2017). Thus, T is a psychobiological mechanism that can influence relationship dynamics between parents, shape how parents allocate limited time and energy to diverse parenting behaviors, and affect the quality of parental engagement in those behaviors (van Anders, 2013; Gettler, 2014). Through these pathways, T has broad implications for family system function as well as child development and well‐being (Boyette, Lew‐Levy, Sarma, & Gettler, 2019; Rosenbaum & Gettler, 2018).

Importantly, the majority of the studies on the psychobiology of partnering and parenting come from samples in cultural settings that emphasize affectionate bonds between parents and that often value warmth and sensitivity in father–child relationships, alongside the mother–child dyad (van Anders, 2013; Gettler, 2016; Rosenbaum & Gettler, 2018). Little is known about parental physiology in settings with different cultural models of family life and parental roles (van Anders, 2013; Gettler, 2014, 2016; Gray, McHale, et al., 2017; Gray, Reece, et al., 2017). However, relevant psychobiological theory and a relatively small number of existing studies suggest that biosocial relationships will vary based on cultural and ecological context (van Anders, 2013; Gettler, 2016). For example, in studies conducted in small‐scale, pastoralist societies in which polygyny is culturally sanctioned, married men and fathers had elevated or comparable T to other men in their populations (Gray, 2003; Muller, Marlowe, Bugumba, & Ellison, 2009). Meanwhile, our team's prior work from the present fisher‐farmer population in Republic of the Congo showed that fathers who were rated as better providers had higher T compared to their peers, which may reflect risk taking or competitive dynamics related to provisioning (Boyette et al., 2019). The relative lack of sampling diversity in this research area limits our ability to build frameworks for the psychobiological, intervention, and clinical implications of these hormones across a broader range of human experience and also poses barriers to framing and reconstructing their adaptive functions in more evolutionarily relevant ecological contexts (Crespi, 2016; Gettler, 2014; Gettler & Oka, 2016; Gettler, Sarma, et al., 2017; Olff et al., 2013; Saxbe, Schetter, Simon, Adam, & Shalowitz, 2017; Swain et al., 2014; van Anders et al., 2011).

Similarly, the scope of psychobiological research on OT and human family dynamics is mostly limited to human populations living in large‐scale, industrialized societies (i.e., generally in the United States, Europe, or other European‐influenced settings; Abraham et al., 2014; Cohen‐Bendahan, Beijers, van Doornen, & de Weerth, 2015; Feldman et al., 2012; Mascaro, Hackett, & Rilling, 2014). Yet, even within this relatively narrow slice human experience, there is increasing evidence that OT can serve diverse social neuroendocrine roles that vary by context (Crespi, 2016; Trumble et al., 2015; van Anders et al., 2011). Among mammals, OT has critical roles in lactation and in the facilitation of mother–offspring bonding, including among humans (Carter, 1998; Rilling & Young, 2014; Trumble et al., 2015). OT appears to have been evolutionarily co‐opted to serve broader psychobiological functions in human females and males, including increasing the salience of both positively and negatively valenced social experiences (Bartz, Zaki, Bolger, & Ochsner, 2011; Bethlehem, Baron‐Cohen, Honk, Auyeung, & Bos, 2014; Crespi, 2016; Feldman, 2012). For example, in the context of nurturing parent–child relationships, mothers and fathers with higher basal OT often engage more sensitively and in greater synchrony with their young children (Feldman, Gordon, Influs, Gutbir, & Ebstein, 2013; Feldman et al., 2012; Gordon, Zagoory‐Sharon, Leckman, & Feldman, 2010). In the only study to date of men's OT in a small‐scale society, Jaeggi, Trumble, Kaplan, and Gurven (2015) showed that among forager‐horticulturalists, men's OT rose across multiple hours of hunting expeditions. They suggested this pattern was consistent with a role for OT reactivity in helping facilitate provisioning and parenting.

Yet, current theory is somewhat unclear regarding how men's provisioning effort in subsistence‐level societies is likely to be linked to OT. Multiple existing models are premised on the idea that heightened OT accompanies and focuses behavior/cognition on socially salient contexts and cues, which can be positively or negatively valenced (Bartz et al., 2011; Bethlehem et al., 2014). In that vein, Crespi (2016) recently proposed a framework that draws on concepts related to human evolution and phylogeny and that is oriented toward integrating the often‐disparate findings for social behavior/cognition and OT, including its links to social support/bonding, anxiety, and outgroup antagonism. In this model, elevated OT during socially salient conditions serves to activate mentalizing networks to enhance social cognition, which can lead to social reward (e.g., in bonded relationships) or heightened anxiety (e.g., in unresolved interpersonal conflict). It is well characterized that in small‐scale societies men who are very successful at securing resources often earn social status in their communities (von Rueden & Jaeggi, 2016); thus, given the social reward of acquired status (von Rueden & Jaeggi, 2016), such fathers could plausibly have elevated OT, based on some psychobiological frameworks (Bartz et al., 2011; Bethlehem et al., 2014; Crespi, 2016).

However, Crespi's (2016) model also emphasizes “joint and opposing effects of [OT] and [T]…(p. 1)” in relationship to human social cognition/behavior, and he goes on to posit that elevated T and reduced OT may be particularly conducive to males' pursuit of physical dominance, which is often a pathway through which men achieve social status in hierarchical small‐scale societies (von Rueden, Gurven, & Kaplan, 2011). In an earlier theoretical contribution, van Anders et al. (2011) influential Steroid/Peptide Theory of Social Bonds specifically focused on integrating the function of multiple psychobiological mechanisms to provide a framework for predictions regarding a range of social behaviors that occur as part of the formation and maintenance of social relationships. They specifically proposed that elevated T and reduced OT within individuals are likely to be coupled to engagement in antagonistic aggression, which they described as involving “obtaining new territory, status, mates, dominance” (van Anders et al., 2011, p. 1268). Drawing from these models, we propose that if men's provisioning effort is largely solitary (i.e., not premised on prosocial bonding and affiliation with peers) and aligns with other behaviors that involve competing with other males for status and dominance (at the expense of social bonding), better providers may have lower basal OT, particularly coupled with elevated T (Crespi, 2016; Trumble et al., 2015; van Anders et al., 2011).

Meanwhile, in romantic relationships, elevated OT has been linked to greater partner support and higher relationship quality and, elsewhere, to heightened anxiety and distress regarding relationship dynamics (Grewen, Girdler, Amico, & Light, 2005; Holt‐Lunstad, Birmingham, & Light, 2015; Marazziti et al., 2006; Taylor et al., 2006; Taylor, Saphire‐Bernstein, & Seeman, 2010). These results highlight the extent to which between‐partner dynamics may correlate with psychobiological functions for OT within the dyad, complementing cross‐partner patterns that have been found for T (Edelstein et al., 2014; Saxbe, Edelstein, et al., 2017). However, these OT‐bonding and OT‐anxiety/distress findings are also potentially contradictory and may be sex‐specific. To date, the latter findings linking higher OT to greater relationship anxiety/distress and trait anxiety (more generally) have been largely observed in women (Marazziti et al., 2006; Taylor et al., 2006, 2010; Weisman, Zagoory‐Sharon, Schneiderman, Gordon, & Feldman, 2013). As such, they point to the need for further research that explores other concomitant neuroendocrine signals within‐individuals to help clarify the psychobiological profiles that emerge under variable relationship and family system conditions. For example, theoretically (van Anders et al., 2011), men and women in nurturant, low‐distress relationships could potentially have profiles of elevated OT and reduced T, while those in relationships characterized by conflict and aggressive behavior would be predicted to have higher T and lower OT. Yet, based on the research reviewed above, that higher T‐lower OT profile may be more common among males, with females potentially exhibiting higher T and higher OT in distressed, conflicted relationships (Edelstein et al., 2014; Marazziti et al., 2006; Taylor et al., 2006, 2010). To date, few studies have considered their joint production within individuals as it relates to the functioning of social relationships that are theoretically relevant to both hormones, such as between parents in dual‐parent family systems (Gordon, Pratt, Bergunde, Zagoory‐Sharon, & Feldman, 2017).

Here, we contribute to this existing literature by examining family roles and conflict, T, and OT in Bondongo mothers and fathers, who reside in a small‐scale, fishing‐farming community in Republic of the Congo. To our knowledge, no prior studies have modeled the dynamics of T, OT, and family life in a small‐scale subsistence‐level society. In our prior psychobiology research from this site, we published results that focused on men's T, fathers' indirect and direct care, and T's correlations with measures of child health. At the time of that publication, we did not have OT data from parents and the earlier work did not include analyses of maternal psychobiology or mother–father conflict (Boyette et al., 2019).

Building from this prior work and because very little is known about paternal OT in small‐scale societies, we first tested whether fisher‐farmer fathers had lower basal OT when they were better providers and had greater conflict with their spouses. To contextualize our research within the broader field of paternal psychobiology, we also tested for relationships between men's OT and direct caregiving. However, we predicted that men's psychobiology would not be robustly linked to this form of parenting, which is much less culturally valued and is relatively uncommon across fathers (Boyette, Lew‐Levy, & Gettler, 2018; Gettler, 2016). We then tested for joint profiles of T and OT within individuals. We predicted that fathers' T would be higher and OT would be lower if they were in marital relationships with greater conflict or they were seen as better providers, respectively. Finally, because past research on family life and labor has shown that household contributions of husbands can affect their wives' psychobiology (Saxbe, Repetti, & Graesch, 2011), we also tested whether fathers' ranks for providing, marital conflict, and direct caregiving predicted maternal T and OT. For example, while average levels of fathers' direct care are minimal in this population, those fathers who were direct caregivers could have reduced the care demands on mothers, contributing to maternal profiles of relatively higher T and lower OT. In a culminating model, we tested whether fathers and mothers exhibited significantly different OT, T profiles from one another based on fathers' roles in the family and marital conflict.

## 2 MATERIALS & METHODS

### 2.1 Study population

As part of a broader project focusing on fathering, family function, and child well‐being, our research team collected data from Bondongo fathers (*n* = 16) and mothers (*n* = 19) in a remote part of northern Republic of the Congo. Descriptive statistics for the sample are reported in Table 1. This Bondongo community is three to six days (via truck and motorboat) from Brazzaville, the capital of the Republic of the Congo. This small‐scale society has a population of <200 total individuals and subsists primarily via fishing and swidden agriculture. Our sample includes 20 total families with at least one child that was less than 18 years of age and represents all eligible participants who were present in the village at the time of data collection. All households had at least one child less than 5 years of age. One father's youngest biological child was 10 years old, but his eldest son coresided in the household and his family had a 2‐month‐old infant (Boyette et al., 2018, 2019). Data collections included demographic, anthropometric (triceps skinfold thickness), and salivary biomarker data, as well as family role ranking measures for fathers (see Section 2.2 below). We collected triceps skinfold thickness data triceps using Lange skinfold calipers by standard techniques (Lohman, Roche, & Martorell, 1988). The study was approved by the Institutional Review Boards of Duke University (Protocol # 2017‐0038) and the University of Notre Dame (# 18‐02‐4397). Individual verbal consent was obtained from all adult participants.

**Table 1:** Descriptive statistics (*n* = 35)

	Men (*n* = 16)		Women (*n* = 19)	
	Mean	*SD*	Mean	*SD*
Age (years)	37.19	8.15	33.67	8.06
Number of children	6.06	3.68	5.26	2.38
Age of youngest child (years)	2.35	2.43	2.22	2.24
Triceps skinfold thickness (mm)^a^	8.81	4.38	22.20	6.49
Fathers' Provider ranking	8.45	3.67	8.39	3.65
Fathers' Dispute ranking	3.11	1.35	2.99	1.27
Fathers' Direct care ranking	4.83	2.01	5.40	2.21
Oxytocin (pg/ml)	44.26	19.76	63.77	26.50
Testosterone (pg/ml)	76.02	36.48	26.40	16.76

a *n* = 17 for women; two females did not have anthropometric data.

### 2.2 Ethnographic context and ranking tasks for familial roles and marital conflict

A major focus of our project was to characterize the way in which the Bondongo participants culturally defined the role of fathers within families (Boyette et al., 2018). After we developed an understanding of their internal cultural perceptions of fatherhood, we then had fathers rank one another according to those characteristics and tested whether those rankings helped explain parental psychobiology. Qualitative ethnographic interviews were conducted with members of the community during initial fieldwork to define the relevant cultural domains, particularly fathers' indirect care (“Provider”: providers of key resources), direct care (“Direct”: e.g., shaping children's proper socialization; caring for ill children), and marital functioning (“Dispute”: conflict with wives, as this negatively affects children). As noted previously (Boyette et al., 2018, 2019), fathers' indirect care was much more highly valued among the Bondongo, compared to direct caregiving. Fathers engaged in relatively little direct care, on average, though with some between‐father variation. As we described recently, men's indirect care activities are physically demanding, often involve risky behavior, and also convey status to men within the community, which has a fairly rigid social hierarchy based on gender, age, and both ascribed and acquired status (Boyette et al., 2019). Females also play important subsistence roles in this community, as they take responsibility for agriculture after men clear the garden plots. Mothers are the primary direct caregivers in this community and receive assistance in direct care from their older children and other kin (e.g., grandparents; Boyette et al., 2018, 2019).

Interrelated with this sex‐based division of labor, Bondongo husband–wife relationships are cooperative and economically productive, but men and women occupy different social and spatial spheres during much of their daily activities. During subsistence tasks (e.g., gardening) and when socializing (such as in the evening), women are typically in the company of other women and their children. Meanwhile, men are more commonly solitary during economic activities and often gather with other adult men to socialize at night. Men generally hold higher status in this patriarchal society; however, women have the ability to divorce husbands and share in decision‐making power within marriages and families, including in terms of reproductive choices. There is a cultural value placed on fertility and large families, which may owe in part to children's contributions to subsistence and household labor. Moreover, at our site and in other similar regional groups (Hewlett, 2013), marital disputes often involve verbal aggression and (less commonly) physical aggression between husbands and wives. Finally, this is a cultural context in which polygyny is culturally sanctioned, though most husbands are partnered to a single wife (Boyette et al., 2018). Our analyses included two families in which men were each polygynously married to two women. In our analyses predicting mothers' hormones, we included all four of these wives. However, we note that if we excluded any combination of these women or removed all four of them from the analyses, the key results for mothers' OT and T profiles were minimally changed (not shown).

For the ranking tasks, fathers were shown photographs of the other participating men in the study and asked to rank their peers in sequence for Provider, Direct, and Dispute, with ties being permissible. This yielded an average peer ranking, from their fellow fathers, for each father on indirect care, direct care, and marital conflict. Men's rankings in each domain showed strong reliability (Cronbach's alphas >0.84) (Boyette et al., 2018, 2019), and they were largely independent of one another (Spearman's *rho*: Provider and Direct = 0.21; Provider and Dispute = 0.16; Direct and Dispute = 0.13; Boyette et al., 2018, 2019). Because of the main focus of this project on culturally defined roles for fathers, we note that we did not collect the same type of peer ranking data from females about mothers nor did we collect mothers' rankings of men in their community.

### 2.3 Salivary T and OT data

Adults provided 2 ml of saliva via passive drool in polypropylene tubes for up to five evenings. The participants came to a centralized location in the village to provide their saliva samples to the research team at the end of each workday. This approach facilitated uniform collection procedures across many participants simultaneously and was also ethically, culturally respectful by enabling individuals to make the choice of whether to participate (or not) on a given day. Because participants arrived at slightly different times, there were typically people providing samples at staggered intervals throughout the sample collection period. This resulted in a regular density of people around the collection location, but this, in and of itself, is not unusual at village gatherings and was a shared social experience across participants during sampling.

The collections began no earlier than 16:30 and generally concluded by 18:30. This represents the early evening period when daytime work concludes, and individuals return from the river and fields to shift their attention toward household tasks. We specifically designed our study to collect repeated evening samples from individuals because T's well‐characterized diurnal curve typically reaches its nadir by late afternoon and remains at that level through the evening, until sleep commences (Gettler, McDade, Feranil, Agustin, & Kuzawa, 2014; Mezzullo et al., 2017; Schmid, Hallschmid, Jauch‐Chara, Lehnert, & Schultes, 2012). This design thus helped our small research team minimize between‐subject and between‐day variability in T that might be attributable to the time of sampling. OT likewise appears to follow a diurnal curve that reaches its nadir by the late afternoon (Forsling, Montgomery, Halpin, Windle, & Treacher, 1998).

Samples were collected within a period of 10 days. Over 60% (men) or 70% (women) of the participants provided 5 samples, with the others providing 3–4 samples each. We collected 159 total saliva samples. Three OT data points and three T data points were excluded from the analyses based on hormone values that were 3+ *SDs* from the mean for the sample. Three OT data points were also below the limit of detection for the assay. In total, our analyses here included 285 total data points for T and OT. Samples were frozen on site at the field location in portable liquid nitrogen dewars and kept frozen until they were transported back to the University of Notre Dame (UND) in a liquid nitrogen dry shipper. The samples were then stored at −80°C at UND until analysis (T) or shipped on dry ice to Arizona State University (OT). Samples were analyzed for T and OT using commercially available kits at UND's Hormones, Health, and Human Behavior Lab (T: Salimetrics, Kit Number: 1‐2402) and by Dr. Benjamin Trumble and colleagues at the CompHEALTH Lab at Arizona State University (OT: Enzo Life Sciences, Kit Number: ADI‐900‐153A). The interassay coefficients of variation (CV) for the low and high controls were as follows: T, 9.6% and 5.6%; OT, 25.9% and 19.3%. The intra‐assay CVs were 4.6% (T) and 11.5%–15.0% (OT). In all relevant analyses, the values for T and OT were base‐10 log‐transformed to adjust for non‐normal distribution of the data. Because the low control CV for OT is higher than ideal, we adjusted the OT values for the assay plate on which they run to remove the effect of between‐plate variability (Reyes et al., 2014). To do so, we regressed the log‐transformed OT values on the plate number, took the residuals of the regression model, and added the mean (of log‐transformed OT) back to the residuals before converting the adjusted OT variable to a *z*‐score.

### 2.4 Covariates

We included theoretically and empirically informed covariates that could confound the relationship between our core predictors (fathers' rankings) and parents' T and OT, respectively. Specifically, we controlled for individuals' ages, total number of children, and age of youngest child. For mothers, we treated age of youngest child as a dichotomous variable to help control for the effects of breastfeeding. Two years of age is approximately the typical weaning age for children in this community, in which all infants are breastfed. Mothers were categorized based on whether they had a child who was two years old or less versus a youngest child who was older than two. We do not have explicit data on breastfeeding frequency/intensity; however, it is well characterized that breastfeeding mothers experience a rapid upward spike in peripheral OT at the initiation of a breastfeeding session and then OT relatively quickly returns to baseline levels after milk letdown (Stuebe, Grewen, & Meltzer‐Brody, 2013). For fathers, we treated age of youngest child as a continuous covariate. Finally, we controlled for the day of sampling in all the linear mixed models (see Section 2.5) and included those results in the Supplemental Materials for our core models (Tables S2–S4).

### 2.5 Statistical analyses

We conducted all statistical analyses using Stata 14.0 (Stata Corporation). The majority of families in the study had full data for both mothers and fathers (*n* = 15 mother–father dyads), but there were a small number of fathers for whom we did not have biological data from mothers and vice versa. Primarily for descriptive purposes, we first ran bivariate correlations between key demographic, anthropometric (triceps skinfold thickness), and ranking variables using Spearman's *rho* (Table 2). By collapsing our repeated measures of OT and T into aggregate means, we would lose substantial individual‐level OT and T information, producing misleading bivariate associations; thus, we did not include the hormones in Table 2. Rather, to show the correlative relationships between participants' OT and T, we used linear mixed models (LMMs) and reported the results in Table 3. We included a measure of anthropometrics in these initial correlative analyses because this is an energetically constrained environment, in which families subsist largely off of natural and cultivated resources in their ecology. We note that adults' triceps skinfold thickness was not significantly correlated to their T or OT, respectively (all *p* > 0.5).

**Table 2:** Bivariate correlations (Spearman's *rho*) between sociodemographics, anthropometrics, and fathers' rankings for family roles

(*n* = 16)	Paternal variables						
	1	2	3	4	5	6	7
Paternal variables							
1. Fathers' # of children	1.0						
2. Age of fathers' youngest child	0.18	1.0					
3. Fathers' triceps skinfolds	0.11	−0.16	1.0				
4. Fathers' age	0.65**	0.27	−0.09	1.0			
5. Fathers' Provider ranking	0.57*	0.25	0.09	0.50*	1.0		
6. Fathers' Dispute ranking	0.10	0.31	−0.26	−0.05	0.16	1.0	
7. Fathers' Direct care ranking	0.57*	−0.06	0.51*	0.39	0.21	0.13	1.0

(*n* = 19)	Maternal variables						
	1	2	3	4	5^a^	6	7
Maternal variables							
1. Mothers' # of children	1.0						
2. Age of mothers' youngest child	0.31	1.0					
3. Mothers' triceps skinfolds^a^	0.13	0.61**	1.0				
4. Mothers' age	0.69**	0.60**	0.47^	1.0			
5. Fathers' Provider ranking	0.52**	0.45^	0.30	0.53*	1.0		
6. Fathers' Dispute ranking	0.35	−0.05	−0.39	0.27	0.10	1.0	
7. Fathers' Direct care ranking	0.50*	0.06	−0.11	0.30	0.13	0.47^	1.0

a *n* = 17; two women did not have anthropometric data.

^^^ *p* < 0.1;

* *p* ≤ 0.05;

** *p* ≤ 0.01.

**Table 3:** Within‐parent associations between oxytocin (OT) and testosterone (T) from linear mixed models^a^

Mothers				Fathers			
Model predicting OT from T				Model predicting OT from T		
*Coef.*	*SE*	*p*		*Coef.*	*SE*	*p*
T	0.02	0.12	0.893	T	0.18	0.17	0.285
	**Model predicting T from OT**				**Model predicting T from OT**		
OT	−0.02	0.10	0.877	OT	0.05	0.08	0.558

a For each model, the hormone variables (OT or T) are log‐transformed values. Models also controlled for day of sampling (not shown).

To model whether men's rankings as fathers predicted their salivary OT and (separately) their wives' salivary OT and T (separately), we used LMMs with maximum‐likelihood estimation. In subsequent analyses testing whether family dynamics predicted joint profiles of T and OT in fathers and mothers, respectively, we again used LMMs with maximum‐likelihood estimation. In the models predicting joint profiles of OT and T, the LMMs included interactions between a categorical variable (“Hormone”) that identified values as T or OT and our key independent variables (IVs: continuous ranking scores for Provider, Direct, and Dispute, respectively). The interaction terms between Hormone and the IVs were modeled because we predicted that T and OT would show opposing joint patterns within individuals (e.g., in families with greater marital conflict, men would have relatively higher T and lower OT, compared to those who dispute less). We included a similar interaction term (Hormone × age of youngest child) for child age, as OT and T could show opposing patterns for that covariate. In each model, we included participant as a random intercept effect. To avoid variance inflation, continuous IVs that were included in interaction terms were centered prior to analysis (Robinson & Schumacker, 2009). For the LMMs, we converted the base‐10 log‐transformed OT and T values to standard scores to ensure that both hormones were on analogous scales and to aid with comparability of model coefficients. In a final culminating analysis, we combined maternal and paternal hormone data into a single LMM that included three‐way interaction terms between Hormone, parent (mother vs. father), and the continuous ranking scores for fathers (Provider, Direct, and Dispute, respectively). This final model allowed us to directly test whether mothers' and fathers' significantly differed for the relationships between fathers' roles and their hormone profiles; the model included a random intercept effect for participant, nested within families.

For the LMMs predicting parents' joint profiles of OT and T, we included figures that visually demonstrate the statistically significant results. We produced the figures using predictive margins in Stata following the LMMs. In those figures, we defined low (−1 *SD* below the mean) and high (+1 *SD* above the mean) ranking scores for the fathers based on the cutoff points in the widely used simple slopes' convention but did so strictly for visual, illustrative purposes. We treated the ranking scores (Provider, Direct, and Dispute) as continuous variables in all our analyses. We evaluated significance at *p* ≤ 0.05.

## 3 RESULTS

As noted above, we reported descriptive statistics for the sample in Table 1 and bivariate correlations between key study variables and relevant covariates in Tables 2 and 3. Mothers were an average of 33.4 years of age (±8.1 *SD*) while fathers were 37.2 years old, on average (±8.2 *SD*). Fathers' total number of children ranged between 1 and 15 (mean: 6.1 ± 3.7 *SD*), while mothers' number of children ranged from 1 to 13 (mean: 5.3 ± 2.4 *SD*). In linear mixed models (LMMs), women had lower T and higher OT than men (both *p* < 0.001; See Table S1a,b), including controlling for age, number of children, and whether the parent had a child two years old or younger. In families in which fathers were ranked as better providers, mothers and fathers had more total children (both *p* < 0.05; Table 2). Older fathers were also viewed as better providers (*p* < 0.01). Fathers and mothers in families in which fathers were seen as more involved with direct care also had more children (both *p* < 0.05). Fathers' OT and T were not significantly associated (*p* > 0.2), nor were mothers' OT and T significantly correlated (*p* > 0.8; Table 3).

**Figure 1:**
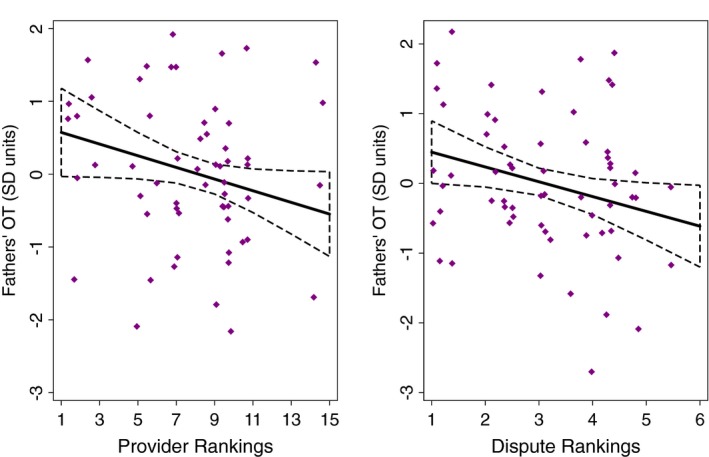
Linear plots (with 95% CI) of fathers' Provider and Dispute rankings predicting their salivary oxytocin (OT) (in *SD* units). The linear plots were derived using predictive margins following the linear mixed model in Table 4. Scatter plots of the standard scores for men's adjusted log OT in relationship to their rankings are overlaid. The log OT standard scores (*n* = 61 from 16 fathers) in the scatter plots were adjusted for the other variables included in Table 4 (beyond men's rankings)

In initial LMMs focused solely on fathers' OT, we found that fathers' who were viewed as having greater conflict with their wives had lower OT (*p* = 0.03; Figure 1; see Tables 4 and S2). Fathers who were ranked as better providers had lower OT than those who were viewed as less effective providers (*p* = 0.05; Figure 1; Table 4). Fathers' direct caregiving (*p* > 0.7) was not significantly linked to their OT. Fathers who were older tended to have lower OT (*p* = 0.055) while those with more children tended to have higher OT (*p* = 0.061; Tables 4 and S2).

**Table 4:** Linear mixed model predicting paternal oxytocin from fathers' rankings for family provisioning, direct caregiving, and marital conflict^a^

	*Coef.*	*SE*	*p*
Provider^b^	−0.08	(0.04)	0.050
Dispute^b^	−0.21	(0.10)	0.030
Direct^b^	−0.02	(0.06)	0.710
Age of youngest child	0.07	(0.07)	0.307
Total # of children	0.08	(0.04)	0.061
Age	−0.04	(0.02)	0.055

a Results reflect analyses of *n* = 61 oxytocin data points from *n* = 16 fathers. Model also controlled for day of sampling (see Table S2).

b Provider: fathers' peer ranking scores for indirect care; Dispute: fathers' peer ranking scores for marital conflict; Direct: fathers' peer ranking scores for direct care.

**Figure 2:**
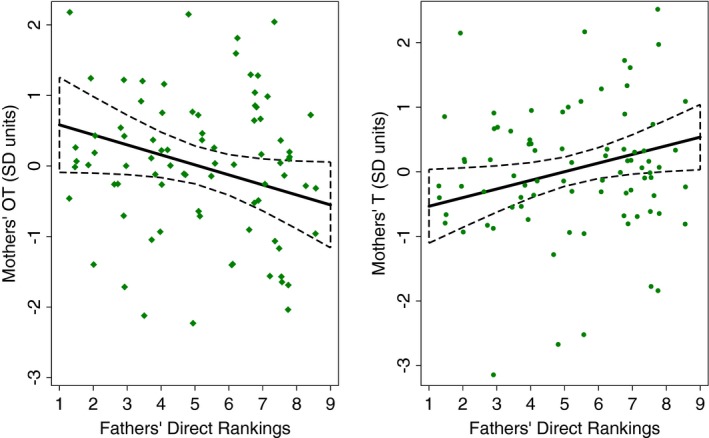
Linear plot (with 95% CI) of fathers' Direct rankings predicting mothers' salivary oxytocin (OT) and testosterone (T) (in *SD* units). The linear plots were derived using predictive margins following the linear mixed model in Table 5. Scatter plots of the standard scores for women's adjusted log OT or adjusted log T in relationship to fathers' Direct rankings are overlaid. The log OT (*n* = 81 from 19 mothers) and log T standard scores (*n* = 83 from 19 mothers) in the scatters plot were adjusted for the other variables included in Table 5

In a similar model for maternal OT, we found that mothers' tended to have higher OT when their spouses were not involved with direct caregiving (*p* = 0.056; Figure 2; see Tables 5 and S3). Mothers' OT did not significantly vary based on fathers' rankings for Provider or Dispute (both *p* > 0.6). In a separate model, mothers also had lower T if fathers were ranked as not engaging in direct care (*p* = 0.032; Figure 2; Tables 5 and S3), while maternal T did not significantly differ based on rankings for Provide and Dispute (both *p* > 0.3). Mothers who had a child that was two years old or less had lower T than mothers whose youngest child was older (*p* = 0.007).

**Table 5:** Linear mixed models predicting maternal oxytocin and testosterone from fathers' rankings for family provisioning, direct caregiving, and marital conflict^a^

	Maternal oxytocin			Maternal testosterone		
	*Coef.*	*SE*	*p*	*Coef.*	*SE*	*p*
Provider^b^	0.03	(0.06)	0.661	0.01	(0.05)	0.793
Dispute^b^	0.02	(0.14)	0.886	−0.12	(0.12)	0.322
Direct^b^	−0.14	(0.07)	0.056	0.13	(0.06)	0.032
Age of youngest child^c^	−0.02	(0.41)	0.970	−0.93	(0.34)	0.007
Total # of children	0.04	(0.09)	0.622	0.09	(0.07)	0.229
Age	0.001	(0.03)	0.982	−0.04	(0.02)	0.085

a Results reflect analyses of *n* = 81 oxytocin data points and *n* = 83 testosterone data points, respectively, from *n* = 19 mothers. Models also controlled for day of sampling (see Table S3).

b Provider: fathers' peer ranking scores for indirect care; Dispute: fathers' peer ranking scores for marital conflict; Direct: fathers' peer ranking scores for direct care.

c For mothers, we treated youngest child as a dichotomous variable indicating whether they had a child who was two years old or less versus a youngest child who was older than two years (comparison group).

**Figure 3:**
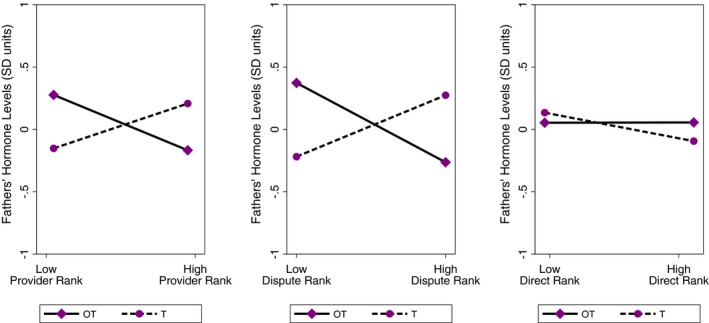
Men's predicted salivary oxytocin (OT) and testosterone (T) based on their rankings for provisioning (Provider), disputing with their wives (Dispute), and direct caregiving (Direct). The plots were derived using predictive margins following the linear mixed model (for fathers) in Table 6. For visual purposes, we present predicted OT and T (in *SD* units) based on fathers having relatively higher (1 *SD* above the mean) or lower (1 *SD* below the mean) scores for Provider, Dispute, and Direct, respectively. We note that we treated rankings for Provider, Dispute, and Direct as continuous variables in the full models in Table 6

In Table 6, we present the full results of LMMs predicting parents' OT and T, separated by sex. For fathers, there were significant interactions for (Hormone × Provider) and (Hormone × Dispute), in models predicting men's OT and T (both *p* < 0.01; also see Table S4). These significant interactions indicate that the slopes for OT and T predicted by men's Provider or Dispute rankings, respectively, are significantly different from one another. As we visually demonstrated in Figure 3, these results both reflected cross‐over interactions. In separate findings for each ranking, fathers' T was relatively higher and their OT lower based on whether they were ranked more highly for Provider or Dispute, respectively (Figure 3). Meanwhile, fathers' T was lower and their OT was higher with lower ranking scores for Provider or Dispute, respectively (Figure 3). The interaction term for men's direct caregiving (Hormone × Direct) was not significant (*p* > 0.3), but the pattern can be seen in Figure 3 for comparison with the results from mothers (below). Fathers' age was a significant covariate (*p* = 0.001; Tables 6 and S4).

**Table 6:** Linear mixed models predicting parental testosterone and oxytocin from fathers' rankings for family provisioning, direct caregiving, and marital conflict

	Model 1. Predicting fathers' testosterone and oxytocin (*n* = 16)^a^			Model 2. Predicting mothers' testosterone and oxytocin (*n* = 19)^a^		
	*Coef.*	*SE*	*p*	*Coef.*	*SE*	*p*
Main effects						
Hormone^b^	−0.65	(0.46)	0.159	0.21	(0.39)	0.586
Provider^c^	−0.06	(0.04)	0.149	0.04	(0.05)	0.407
Dispute^c^	−0.24	(0.13)	0.027	0.07	(0.12)	0.557
Direct^c^	0.08	(0.07)	0.257	−0.13	(0.06)	0.038
Age of youngest child^d^	0.10	(0.07)	0.184	−0.04	(0.06)	0.915
Total # of children	0.07	(0.04)	0.082	0.07	(0.07)	0.287
Age	−0.05	(0.02)	0.001	−0.02	(0.02)	0.332
Interaction terms						
Provider × Hormone	0.11	(0.04)	0.009	−0.04	(0.05)	0.331
Dispute × Hormone	0.43	(0.13)	0.001	−0.24	(0.11)	0.035
Direct × Hormone	−0.08	(0.08)	0.335	0.26	(0.06)	<0.001
Age of youngest child × Hormone	−0.03	(0.09)	0.687	−0.85	(0.35)	0.019

a Results for Model 1 reflect analyses of *n* = 121 hormone data points from *n* = 16 fathers. Results for Model 2 reflect analyses of *n* = 164 hormone data points from *n* = 19 mothers. Models also controlled for day of sampling (see Table S4).

b Hormone: a dichotomous variable indicating whether the dependent variable data point is oxytocin or testosterone.

c Provider: fathers' peer ranking scores for indirect care; Dispute: fathers' peer ranking scores for marital conflict; Direct: fathers' peer ranking scores for direct care.

d We treated age of youngest child as a continuous variable for fathers. For mothers, we treated youngest child as a dichotomous variable indicating whether they had a child who was two years old or less versus a youngest child who was older than two years (comparison group).

**Figure 4:**
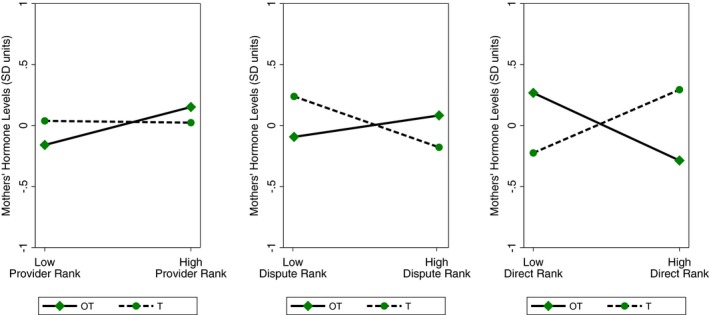
Women's predicted salivary oxytocin (OT) and testosterone (T) based on their husbands' rankings for provisioning (Provider), marital disputing (Dispute), and direct caregiving (Direct). The plots were derived using predictive margins following the linear mixed model (for mothers) in Table 6. For visual purposes, we present predicted OT and T (in *SD* units) based on whether mothers' husbands had relatively higher (1 *SD* above the mean) or lower (1 *SD* below the mean) scores Provider, Dispute, and Direct, respectively. We note that we treated rankings for Provider, Dispute, and Direct as continuous variables in the full models in Table 6

For mothers, we found significant cross‐over interactions for (Hormone × Direct) and (Hormone × Dispute), respectively, in models predicting maternal hormone values (both *p* < 0.05). See Tables 6 and S4 for full results. As shown in Figure 4, mothers' OT was relatively higher and their T was lower if their spouses were rated as being less involved with direct care (Direct). Maternal OT was comparatively lower and their T was higher if their partners were ranked as doing more direct care. Notably, the cross‐over interaction for mothers based on fathers' Dispute rankings showed the opposite pattern to that of fathers (see Figure 4). Across mothers, OT was higher and T was lower if they were in marriages with more marital conflict, although the slopes were relatively modest. In marriages in which mothers and fathers were perceived to have less conflict, maternal OT was relatively lower and T was higher (Figure 4). The interaction term for (Hormone × age of youngest child) was a significant covariate (*p* = 0.019).

Finally, in a culminating three‐way interaction model, we tested whether parent (mother vs. father) significantly moderated the relationship between Hormone and fathers' rankings (e.g., Hormone × Dispute × parent). As shown in Table 7, the respective three‐way interaction terms for Provider, Dispute, and Direct were all statistically significant (all *p* < 0.05). These results indicate that the divergent profiles for maternal and paternal OT and T, based on fathers' rankings, shown in Figures 3 and 4 were significantly different from one another.

**Table 7:** Linear mixed models predicting parental testosterone and oxytocin from fathers' rankings for family provisioning, direct caregiving, and marital conflict (*n* = 35)^a^

	Coef.	*SE*	*p*
Main effects			
Hormone^b^	0.06	(0.05)	0.198
Provider^c^	0.07	(0.36)	0.851
Dispute^c^	0.10	(0.11)	0.349
Direct^c^	−0.12	(0.06)	0.045
Age of youngest child^d^	−0.08	(0.34)	0.815
Total # of children	0.06	(0.03)	0.082
Age	−0.03	(0.01)	0.008
Parent	0.40	(0.35)	0.255
Interaction terms			
Provider × Hormone × parent	0.16	(0.06)	0.015
Dispute × Hormone × parent	0.61	(0.17)	<0.001
Direct × Hormone × parent	−0.36	(0.10)	0.001
Age of youngest child × Hormone × parent	0.99	(0.53)	0.060

a Results reflect analyses of *n* = 285 hormone data points from *n* = 16 fathers and *n* = 19 mothers. Given the three‐way interaction terms are the primary focus of this model, we do not report the results for the two‐way interaction terms. The more relevant sex‐specific two‐way interaction terms can be found in Table 5. The model also controlled for day of sampling (not shown).

b Hormone: a dichotomous variable indicating whether the dependent variable data point is oxytocin or testosterone.

c Provider: fathers' peer ranking scores for indirect care; Dispute: fathers' peer ranking scores for marital conflict; Direct: fathers' peer ranking scores for direct care.

d We treated youngest child as a dichotomous variable indicating whether they had a child who was two years old or less versus a youngest child who was older than two years (comparison group).

## 4 DISCUSSION

A primary aim of the current analyses was to test for joint profiles of T and OT within mothers and fathers based on measures of family system dynamics, including culturally informed measures of fathers' contributions to family life and marital conflict. As we outlined in the Introduction, there is a substantial body of research on men's T, life history status, and fathering (Gettler, 2014; Gray, Reece, et al., 2017; van Anders, 2013). Comparatively, there is less research on fathers' OT and mothers' T, and little prior work has considered their joint production within parents in relationship to family dynamics (Gordon et al., 2017). A second complementary contribution of this work is that we explored these issues in the context of a small‐scale, subsistence‐level society in the Republic of the Congo. The vast majority of the research on human paternal and maternal psychobiology has been conducted in societal settings in which sensitive, nurturant, and/or warm parent–parent and father–child relationships are emphasized (Feldman, 2012; Gettler, 2014, 2016; van Anders, 2013). Given the importance of OT and T to parenting/partnering behaviors as well as parental mental and physical health, understanding the range of their expression across human contexts will be informative to models of family system function and child outcomes.

### 4.1 Fathers' OT and joint profiles for OT and T

Bondongo men had significantly higher T and lower OT than women. The male–female differences for T are unsurprising. However, the sex differences for OT stand out from prior research on OT assayed from saliva and other media (plasma; urine) that have found no male–female differences (Feldman, Gordon, Schneiderman, Weisman, & Zagoory‐Sharon, 2010; Feldman, Gordon, & Zagoory‐Sharon, 2011; Feldman et al., 2012; Grewen, Davenport, & Light, 2010; Taylor et al., 2010) or, in one large study, that Israeli men's OT was higher than women's (Weisman et al., 2013). While it is plausible that our inability to directly control for breastfeeding status (see Section 4.3 below) contributed to this finding, the sex difference persisted even if women with breastfeeding‐aged children were eliminated (see Table S1b). We are hesitant to speculate too broadly regarding the factors that might contribute to male–female OT differences among the Bondongo that are distinct from patterns in populations from settings such as the United States, Israel, and Europe. It seems plausible that the variation could be associated with cultural differences in social hierarchy and/or gender roles, meriting consideration in future comparative research.

Among Bondongo fathers, those who were seen as having greater conflict with their spouses had lower OT than those who disputed less with their wives. We observed a complementary, significant cross‐over interaction for their joint profiles of T and OT. Fathers exhibited relatively higher T and lower OT if they were perceived to have greater conflict with their wives. In contrast, fathers who were in marriages with less discord had relatively elevated OT and lower T. Our results complement prior work from other settings in which men had elevated OT when they felt better supported by their partners and reported higher relationship quality (Grewen et al., 2005; Holt‐Lunstad et al., 2015). In addition, U.S. men with lower T reported being more satisfied and committed to their relationships and had less marital conflict and higher marital quality, particularly under stressful family conditions (Booth & Dabbs, 1993; Booth, Johnson, & Granger, 2005; Edelstein et al., 2014). Among the Bondongo, the community is hierarchical and men are considered dominant to women in terms of status. Husbands and wives can also be verbally and (less commonly) physically aggressive toward their partners. Thus, men's within‐individual profiles of relatively elevated T and reduced OT in conflicted marriages also conceptually align with psychobiological research linking higher T to reactive responses to status threats as well as lower OT to reduced empathy (Crespi, 2016; Feldman, 2012; Wagels et al., 2018; Wirth & Schultheiss, 2007).

Bondongo fathers also had lower OT when their peers ranked them as better providers. Moreover, in our interaction models, fathers seen as better providers had relatively elevated T and lower OT compared to men who were seen as less effective providers, who had the opposing profile of relatively higher OT and lower T. As we have described in previous work from this population, men's subsistence tasks in this setting often involve risky behaviors (e.g., climbing high trees for resources; facing dangers while fishing, including drowning risks and threats from animals, like crocodiles). Men's provisioning roles also represent social mechanisms through which they acquire status in the community (Boyette et al., 2019). Thus, these represent potential psychobiological pathways linking elevated T and provisioning among Bondongo fathers, as risk taking behavior and competition for status are both social–behavioral domains that have been linked to elevated T in humans and other primates (Boyette et al., 2019; Gray, Reece, et al., 2017; Muller, 2017). For fathers' OT, past observational research on Israeli families showed that men with elevated OT were more affectionately and positively engaged when they interacted with their infants (Feldman et al., 2012, 2013; Gordon et al., 2010). Among U.S. fathers, exogenous intranasal OT enhanced fathers' neural responses to photographs of their own children in brain areas relevant to reward and attention (Li, Chen, Mascaro, Haroon, & Rilling, 2017). In similar cultural settings, fathers receiving exogenous OT engaged in positive behaviors with their young children, such as encouragement and stimulation of exploration (Naber, van Ijzendoorn, Deschamps, van Engeland, & Bakermans‐Kranenburg, 2010; Weisman, Zagoory‐Sharon, & Feldman, 2012).

A collective perspective on this past work suggests that in societal contexts and families in which paternal sensitivity and nurturing are common, fathers performing such roles will tend to have lower T and elevated OT (van Anders, 2013; van Anders et al., 2011; Gettler, 2014). To our knowledge, only one prior study has attempted to link fathers' parenting with their dual profiles for endogenous OT and T. Gordon and colleagues (2017) showed that Israeli fathers with elevated OT and lower T engaged in greater levels of affectionate touch with their infants during home observations, compared to men with other OT‐T profiles. Here, we did not find statistically significant links between men's direct caregiving rankings and their OT (singularly) or their within‐individual profiles for OT and T. In particular, this stands in contrast to the more robust pattern for Bondongo fathers' (culturally valued) indirect care, as those who were ranked highly for provisioning exhibited profiles of relatively higher T and lower OT.

In one of the only prior naturalistic studies of OT in a small‐scale society that emphasizes fathers' provisioning, Jaeggi et al. (2015) found that Tsimane foragers' salivary OT increased across a multiple‐hour hunting expedition. In this study, men who exhibited greater OT increases during the hunt likewise showed larger elevations in T. While these findings ostensibly conflict with the patterns we documented among Bondongo fathers, they are potentially congruent because of the distinction between basal hormone production and shorter‐term reactivity (Kuo et al., 2018; Trumble et al., 2015). Based on existing models (van Anders et al., 2011; Crespi, 2016), we suggest it is plausible that in socially hierarchical contexts such as among the Bondongo and Tsimane (von Rueden et al., 2011), men who are ranked highly for dominance and status will tend to have elevated basal T and reduced basal OT, but will show short‐term rises in OT production in response to socially rewarding contexts pertaining to status (e.g., hunting for Tsimane men). The potential complementarity of basal and reactive OT functions in this model is similar to arguments made regarding such basal/reactive divergences for males' T, including based on behavioral and biological costs of maintaining chronically elevated basal T (Roney & Gettler, 2015; Storey, Walsh, Quinton, & Wynne‐Edwards, 2000; Trumble et al., 2013). While our idea is speculative at this time, these coconsiderations of basal levels and acute reactivity merit further attention in future research on fathers' OT and family roles, paralleling past work on hormones such as T, cortisol, and prolactin (Fleming, Corter, Stallings, & Steiner, 2002; Kuo et al., 2018).

### 4.2 Mothers' OT and T

The psychobiological functions of maternal OT and T are also relatively understudied cross‐culturally, particularly in small‐scale societies and cultures with different models of family life, compared to the United States, Europe, and related contexts. Reflecting a modest cross‐over interaction in our models, Bondongo mothers' who were in marriages with higher levels of conflict had relatively modestly elevated OT and lower T. That is the opposing hormone profile of what we observed in Bondongo fathers in comparable marriages, and the maternal and paternal patterns differed significantly from one another. These results indicate that Bondongo husbands and wives exhibit sex‐specific psychobiological patterns in marriages with greater discord. Multiple social neuroendocrine studies of European and U.S. females found that women with higher OT reported elevated romantic relationship anxiety and distress and lower relationship quality (Marazziti et al., 2006; Taylor et al., 2006, 2010). Moreover, Taylor et al., 2010 found that men did not exhibit similar positive correlations between OT and relationship distress, complementing the divergent profiles we observed for Bondongo husbands and wives. The male–female differences in our study could reflect gender socialization and roles for social behavior.

Women are the primary caregivers and family nurturers in Bondongo society. Thus, compared to men, the task of ameliorating marital relationship distress and the burden of anxiety when such conflict occurs may fall more heavily on females, which might then be reflected in their psychobiological profiles of relatively lower T and higher OT (van Anders et al., 2011; Ditzen et al., 2012; Grebe et al., 2017; Taylor et al., 2006). Because our data are correlational, we cannot determine the direction of the effects between maternal psychobiology and participation in marriages with conflict. Thus, one additional possibility is that women with certain psychobiological profiles (e.g., lower OT, higher T) have behavioral dispositions that are associated with less marital conflict (i.e., rather than mothers' OT‐T being shaped by conflict experiences). For example, Bondongo women can also be verbally and (occasionally) physically aggressive toward their husbands. It is plausible that women with relatively lower OT and higher T are more effective at responding to challenges from their partners and at establishing greater equity and lower conflict within the dyad. These questions of directionality merit exploration in future longitudinal research, as has been conducted for paternal psychobiology elsewhere (Edelstein et al., 2017; Gettler, Ryan, et al., 2017; Saxbe, Edelstein, et al., 2017).

We also found that Bondongo mothers had higher OT and lower T, respectively, when their partners were seen as less involved with direct caregiving, which is theoretically consistent with heightened maternal roles as nurturant caregivers in this setting (van Anders et al., 2011; Feldman, 2012). Those single‐hormone patterns parallel the significant cross‐over interaction we observed for maternal profiles for OT and T based on fathers' direct care rankings. Bondongo fathers perform relatively little direct care, on average, and that role for fathers is not as culturally valued as their contributions as providers. Yet, we have previously shown that fathers who were ranked highly as direct caregivers had healthier children at this site, and their direct care was more strongly linked to child health than men's ranks as providers (Boyette et al., 2018, 2019). Thus, our findings here suggest another way in which fathers' direct care is correlated with aspects of family system function.

Prior research found that mothers with young children requiring intensive care had lower T than other women (Barrett et al., 2013; Kuzawa et al., 2010). However, somewhat contrasting with our results, Gordon et al. (2017) showed that Israeli mothers with higher OT and elevated T were more affectionate during interactions with their infants. For Bondongo families in which fathers were ranked highly as direct caregivers, mothers' higher T and lower OT could be reflective of comparatively reduced caregiving demands (also see Section 4.3 below). From a family systems perspective, it is also plausible that fathers' heightened direct caregiving in such families is a response to maternal disposition, including mothers' psychobiological profiles. This possibility also conceptually aligns with Gordon et al., 2017 findings for lower OT, higher T Israeli mothers, who were less affectionate. Given our cross‐sectional data, we cannot resolve these issues but they are worthwhile questions for future research.

### 4.3 Limitations

Our study has a number of limitations that are worthy of discussion. First, our sample sizes for mothers and fathers were relatively small compared to studies of maternal and paternal psychobiology in industrialized settings in more highly populated societies (Barrett et al., 2013; Feldman et al., 2012; Gettler, Sarma, et al., 2017; Gordon et al., 2017; Kuo et al., 2018). Small sample sizes limit statistical power but can also contribute to inflated effect sizes for statistically significant results (Button et al., 2013). One of the important contributions of our study is the focus on these psychobiological dynamics in a small‐scale, subsistence‐level society setting, which differs politically, economically, and culturally from sites of prior work on this topic (Gettler, 2016). However, as we have discussed in our past work from this site, smaller sample sizes are a research design trade‐off that result from working at a highly remote field site with participants residing in a small community (Boyette et al., 2019). We note that we collected data from nearly all the eligible, present members of the group at the time of the study, and our total sample of 35 adults encompasses at least one member of every family in this community. Moreover, we attempted to address some of these concerns regarding overall sample size by conducting repeated sampling across individuals for up to five days, yielding a total 285 data points for OT and T in our analyses. We also used data analytical techniques that maximized the information from these repeated observations.

In addition, our study was cross‐sectional and correlative, which similarly limits statistical power and the scope of our conclusions. Specifically, we cannot speak to the direction of the effects between fathers' roles in Bondongo families and parental psychobiology. There are plausible bidirectional relationships between fathering, family system function, and both T and OT, respectively, which we have attempted to succinctly describe above. In the future, we aim to collect longitudinal data at this site, which would help us document within‐individual changes in perceptions of fathers' roles as well as maternal and paternal hormones.

There are also methodological limitations of our work that pertain to the measurement of salivary OT and T. First, we measured OT peripherally via saliva. Peripheral OT is released from the posterior pituitary into circulation and then cannot readily cross the blood–brain barrier. Meanwhile, central release of OT is the primary mechanism through which OT affects behavior, cognition, and emotion (Carter, 1998; Feldman, 2012; Gettler, 2014; Rilling & Young, 2014). Rodent studies suggest that peripheral and central OT release occurs simultaneously in response to some social and physical stimuli while they are decoupled in response to others (Landgraf & Neumann, 2004). Here, we found multiple patterns for joint profiles of OT and T in mothers and fathers that are consistent with current psychobiological frameworks and align with neuroendocrine functions that could parallel central OT (Feldman, 2012; Gettler, 2014; Trumble et al., 2015; van Anders, 2013; van Anders et al., 2011). However, because of the limitations of peripheral OT measurements, we cannot interpret our nonsignificant findings for Bondongo parents as evidence that OT has minimal contributing roles in those parenting domains. One additional methodological concern regarding the OT data is that we did not collect systematic behavioral observations during sample collection, particularly whether women were breastfeeding while providing saliva. We controlled for whether women had a child under weaning age to attempt to mitigate this issue. Moreover, the largest effect we observed for women's OT was such that their OT was higher when their spouses were perceived as doing less direct care. In this sample, fathers' direct caregiving rankings were not meaningfully correlated with the age of their youngest child, including whether they had a child under weaning age. So, we think it is highly unlikely that women's nursing status was an unmeasured confounder of the correlation between maternal OT and fathers' direct caregiving.

Briefly, we also note that concerns have been raised regarding measurement of OT in saliva. Observational, experimental, and pharmacological research help support the validity of salivary OT as an index of peripheral OT function (de Jong et al., 2015; Grewen et al., 2010; Martin et al., 2018; Weisman et al., 2012). Moreover, salivary OT is often at least moderately correlated with plasma levels, and recent research also showed that salivary OT was a stronger indicator of cerebrospinal fluid (CSF) OT, compared to plasma OT, giving us further confidence in our approach (Martin et al., 2018). A further limitation of our study's OT data pertains to the relatively elevated interassay coefficient of variation for the low control values. To reduce chance effects of between‐plate variability on our outcomes of interest, we adjusted the OT values for their plate of origin before our core analyses. Moreover, between‐plate variability does not bias the OT data one way or another (i.e., higher or lower) but rather reduces data reliability, which should limit statistical power and increase the likelihood of Type II error.

Additionally, recent assay methodological research has helped shed light on accuracy issues for salivary T in the female range when measured using ELISA techniques, as we did here (Welker et al., 2016). The work by Welker and colleagues (2016) showed that ELISA approaches tended to inflate T values for particularly low salivary T levels, as are commonly found in women. We agree with these researchers and others that studies aiming to measure salivary T, especially in women, should move toward use of liquid chromatography tandem mass spectrometry (Mazur & Clifton, 2018; Welker et al., 2016). While it is important to address these caveats to our results, we also think it is unlikely that the potential inflation of lower T values for females in this community would specifically help explain our key results for maternal psychobiology given this issue should reduce the reliability of the data and that many of the core findings were based on cross‐over interactions between T and OT, including comparisons with fathers.

Finally, we note that a limitation of our findings is that we only had rankings of fathers' family roles in this community, rather than measures from a broader array of family members, including mothers. This limited our ability to draw specific conclusions about the factors shaping maternal psychobiology. For example, we found that fathers' direct care rankings predicted mothers' psychobiology, but we can only hypothesize that mothers are able to do less direct care in families in which fathers do more. It is also plausible that unmeasured confounding variables could help explain those patterns, such as families having varying degrees of assistance from other alloparental caregivers like grandmothers and older siblings that correlate with fathers' roles. In future work from this site, we hope to collect thorough interview and systematic observational data from an array of family members, rather than just fathers.

## 5 CONCLUSIONS

In this study, we drew on social neuroendocrine perspectives to test how family roles related to fathers' OT, mothers' OT and T, and joint profiles of OT and T within parents in a small‐scale, subsistence‐level society. Bondongo fathers had relatively higher T and lower OT if they were seen as better providers. In this socially hierarchical society in which men compete for status, including as providers, these novel results link fathers' dual profiles for OT and T to such roles, rather than solely to nurturant caregiving as in most prior work in this area. Bondongo mothers' within‐individual profiles for OT and T also differed based on fathers' direct caregiving and marital conflict, respectively. In families with high degrees of husband–wife conflict, we found that mothers and fathers had opposing profiles of T and OT, which highlights the cross‐partner effects of marital difficulties and also the sex‐based differences in their psychobiological correlates in this setting. Aligning with current theoretical frameworks (Gettler, 2016; van Anders, 2013; van Anders et al., 2011), these findings help to shed light on the flexibility of human paternal and maternal psychobiology when fathers' roles and functions within families differ across cultures.

## CONFLICT OF INTEREST

The authors declare no conflicts of interest.

## Data Availability

The data that support the findings of this study are available from the corresponding author upon reasonable request.
