# Supplementary material for: Mothers' and fathers' joint profiles for testosterone and oxytocin in a small‐scale fishing‐farming community: Variation based on marital conflict and paternal contributions
Source: Brain Behav. 2019 Aug 5;9(9):e01367. doi: 10.1002/brb3.1367 (PMC6749485; doi:10.1002/brb3.1367)
Supplement: Supplementary file 1 [file BRB3-9-e01367-s001.docx]

| **Supp. Table 1a. Linear mixed models testing for sex differences in parental oxytocin and testosterone**^†^ | | | | | | | |
| --- | --- | --- | --- | --- | --- | --- | --- |
|  | **oxytocin** | | |  | **testosterone** | | |
|  | *coef.* | *SE* | *p* |  | *coef.* | *SE* | *p* |
| male^‡^ | -0.13 | (0.04) | <0.001 |  | 0.52 | (0.05) | <0.001 |
| age of youngest child^¶^ | -0.04 | (0.04) | 0.237 |  | -0.17 | (0.06) | 0.003 |
| total # of children | -0.003 | (0.003) | 0.275 |  | -0.01 | (0.004) | 0.040 |
| age | 0.003 | (0.01) | 0.609 |  | 0.02 | (0.01) | 0.068 |
| day 2 | -0.14 | (0.06) | 0.018 |  | -0.04 | (0.06) | 0.489 |
| day 3 | -0.07 | (0.05) | 0.148 |  | -0.08 | (0.05) | 0.128 |
| day 4 | -0.18 | (0.05) | 0.001 |  | -0.08 | (0.06) | 0.174 |
| day 5 | -0.09 | (0.06) | 0.128 |  | -0.02 | (0.06) | 0.801 |
| day 6 | -0.04 | (0.06) | 0.495 |  | -0.01 | (0.06) | 0.893 |
| day 7 | -0.15 | (0.05) | 0.006 |  | -0.13 | (0.06) | 0.030 |
| day 8 | -0.20 | (0.06) | <0.001 |  | -0.03 | (0.06) | 0.652 |
| day 9 | -0.14 | (0.05) | 0.011 |  | -0.09 | (0.06) | 0.142 |
| **Supp. Table 1b. Linear mixed models testing for sex differences in parental oxytocin and testosterone, excluding mothers with children ≤ 2 years of age**^†^ | | | | | | | |
|  | **oxytocin** | | |  | **testosterone** | | |
|  | *coef.* | *SE* | *p* |  | *coef.* | *SE* | *p* |
| male^‡^ | -0.19 | (0.04) | <0.001 |  | 0.44 | (0.07) | <0.001 |
| age of youngest child^¶^ | 0.003 | (0.04) | 0.941 |  | -0.10 | (0.07) | 0.141 |
| total # of children | -0.004 | (0.002) | 0.058 |  | -0.01 | (0.004) | 0.008 |
| age | 0.004 | (0.004) | 0.452 |  | 0.02 | (0.01) | 0.041 |
| day 2 | -0.19 | (0.07) | 0.004 |  | -0.08 | (0.07) | 0.267 |
| day 3 | -0.09 | (0.06) | 0.116 |  | -0.07 | (0.06) | 0.246 |
| day 4 | -0.20 | (0.06) | 0.001 |  | -0.09 | (0.07) | 0.199 |
| day 5 | -0.16 | (0.07) | 0.015 |  | -0.03 | (0.07) | 0.683 |
| day 6 | -0.12 | (0.09) | 0.157 |  | 0.10 | (0.09) | 0.261 |
| day 7 | -0.23 | (0.06) | <0.001 |  | -0.09 | (0.08) | 0.234 |
| day 8 | -0.23 | (0.06) | <0.001 |  | 0.00 | (0.07) | 0.979 |
| day 9 | -0.18 | (0.07) | 0.005 |  | -0.02 | (0.08) | 0.793 |

^†^Supp. Table 1a results reflect analyses of n = 142 oxytocin data points and n = 143 testosterone data points, respectively, from n = 19 mothers and n = 16 fathers. Supp. Table 1b results reflect analyses of n = 92 oxytocin data points and n = 92 testosterone data points, respectively, from n = 7 mothers and n = 16 fathers. For each model, the hormone variables (oxytocin or testosterone) are log-transformed values.

^‡^male: dichotomous variable comparing males to females.

^¶^We treated youngest child as a dichotomous variable indicating whether they had a child who was two years old or less versus a youngest child who was older than two years (comparison group).

| **Supp. Table 2. Linear mixed model predicting paternal oxytocin from fathers’ rankings for family provisioning, direct caregiving, and marital conflict**^†^ | | | | |
| --- | --- | --- | --- | --- |
|  | *coef.* | *SE* | *p* |  |
| Provider^‡^ | -0.08 | (0.04) | 0.050 |  |
| Dispute^‡^ | -0.21 | (0.10) | 0.030 |  |
| Direct^‡^ | -0.02 | (0.06) | 0.710 |  |
| age of youngest child | 0.07 | (0.07) | 0.307 |  |
| total # of children | 0.08 | (0.04) | 0.061 |  |
| age | -0.04 | (0.02) | 0.055 |  |
| day 2 | -1.16 | (0.48) | 0.016 |  |
| day 3 | -0.17 | (0.41) | 0.682 |  |
| day 4 | -1.05 | (0.46) | 0.024 |  |
| day 5 | -1.12 | (0.46) | 0.016 |  |
| day 6 | -0.62 | (0.68) | 0.361 |  |
| day 7 | -1.25 | (0.43) | 0.004 |  |
| day 8 | -1.38 | (0.45) | 0.002 |  |
| day 9 | -1.31 | (0.50) | 0.010 |  |

^†^Results reflect analyses of n = 61 oxytocin data points from n = 16 fathers.

^‡^Provider: fathers’ peer ranking scores for indirect care; Dispute: fathers’ peer ranking scores for marital conflict; Direct: fathers’ peer ranking scores for direct care.

| **Supp. Table 3. Linear mixed models predicting maternal oxytocin and testosterone from fathers’ rankings for family provisioning, direct caregiving, and marital conflict**^†^ | | | | | | | |
| --- | --- | --- | --- | --- | --- | --- | --- |
|  | **maternal oxytocin** | | |  | **maternal testosterone** | | |
|  | *coef.* | *SE* | *p* |  | *coef.* | *SE* | *p* |
| Provider^‡^ | 0.03 | (0.06) | 0.661 |  | 0.01 | (0.05) | 0.793 |
| Dispute^‡^ | 0.02 | (0.14) | 0.886 |  | -0.12 | (0.12) | 0.322 |
| Direct^‡^ | -0.14 | (0.07) | 0.056 |  | 0.13 | (0.06) | 0.032 |
| age of youngest child^¶^ | -0.02 | (0.41) | 0.970 |  | -0.93 | (0.34) | 0.007 |
| total # of children | 0.04 | (0.09) | 0.622 |  | 0.09 | (0.07) | 0.229 |
| age | 0.001 | (0.03) | 0.982 |  | -0.04 | (0.02) | 0.085 |
| day 2 | -0.52 | (0.41) | 0.207 |  | 0.03 | (0.30) | 0.913 |
| day 3 | -0.61 | (0.34) | 0.072 |  | -0.23 | (0.26) | 0.379 |
| day 4 | -1.13 | (0.38) | 0.003 |  | -0.41 | (0.28) | 0.154 |
| day 5 | -0.27 | (0.45) | 0.549 |  | -0.18 | (0.31) | 0.564 |
| day 6 | -0.21 | (0.39) | 0.578 |  | -0.12 | (0.28) | 0.656 |
| day 7 | -0.66 | (0.38) | 0.081 |  | -0.54 | (0.30) | 0.068 |
| day 8 | -1.16 | (0.45) | 0.009 |  | 0.01 | (0.34) | 0.980 |
| day 9 | -0.64 | (0.38) | 0.088 |  | -0.44 | (0.29) | 0.135 |

^†^Results reflect analyses of n = 81 oxytocin data points and n = 83 testosterone data points, respectively, from n = 19 mothers.

^‡^Provider: fathers’ peer ranking scores for indirect care; Dispute: fathers’ peer ranking scores for marital conflict; Direct: fathers’ peer ranking scores for direct care.

^¶^For mothers we treated youngest child as a dichotomous variable indicating whether they had a child who was two years old or less versus a youngest child who was older than two years (comparison group).

| **Supp. Table 4. Linear mixed models predicting parental testosterone and oxytocin from fathers’ rankings for family provisioning, direct caregiving, and marital conflict** | | | | | | | |
| --- | --- | --- | --- | --- | --- | --- | --- |
|  | **Model 1.**  **predicting fathers’**  **testosterone and oxytocin**  **(n = 16)^†^** | | |  | **Model 2.**  **predicting mothers’**  **testosterone and oxytocin**  **(n = 19)^†^** | | |
|  | *coef.* | *SE* | *p* |  | *coef.* | *SE* | *p* |
| **main effects** |  |  |  |  |  |  |  |
| Hormone^‡^ | -0.65 | (0.46) | 0.159 |  | 0.21 | (0.39) | 0.586 |
| Provider^§^ | -0.06 | (0.04) | 0.149 |  | 0.04 | (0.05) | 0.407 |
| Dispute^§^ | -0.24 | (0.13) | 0.027 |  | 0.07 | (0.12) | 0.557 |
| Direct^§^ | 0.08 | (0.07) | 0.257 |  | -0.13 | (0.06) | 0.038 |
| age of youngest child^¶^ | 0.10 | (0.07) | 0.184 |  | -0.04 | (0.06) | 0.915 |
| total # of children | 0.07 | (0.04) | 0.082 |  | 0.07 | (0.07) | 0.287 |
| age | -0.05 | (0.02) | 0.001 |  | -0.02 | (0.02) | 0.332 |
| day 2 | -1.13 | (0.47) | 0.016 |  | -0.51 | (0.38) | 0.176 |
| day 3 | -0.13 | (0.40) | 0.751 |  | -0.67 | (0.31) | 0.033 |
| day 4 | -0.88 | (0.46) | 0.054 |  | -1.14 | (0.35) | 0.001 |
| day 5 | -0.97 | (0.46) | 0.035 |  | -0.26 | (0.41) | 0.520 |
| day 6 | -0.57 | (0.67) | 0.398 |  | -0.22 | (0.35) | 0.528 |
| day 7 | -1.24 | (0.43) | 0.004 |  | -0.67 | (0.35) | 0.057 |
| day 8 | -1.29 | (0.44) | 0.004 |  | -1.21 | (0.41) | 0.003 |
| day 9 | -1.23 | (0.50) | 0.014 |  | -0.66 | (0.34) | 0.055 |
| **interaction terms** |  |  |  |  |  |  |  |
| Provider × Hormone | 0.11 | (0.04) | 0.009 |  | -0.04 | (0.05) | 0.331 |
| Dispute × Hormone | 0.43 | (0.13) | 0.001 |  | -0.24 | (0.11) | 0.035 |
| Direct × Hormone | -0.08 | (0.08) | 0.335 |  | 0.26 | (0.06) | <0.001 |
| age of youngest child × Hormone | -0.03 | (0.09) | 0.687 |  | -0.85 | (0.35) | 0.019 |
| day 2 × Hormone | 0.79 | (0.64) | 0.217 |  | 0.58 | (0.51) | 0.261 |
| day 3 × Hormone | -0.28 | (0.56) | 0.619 |  | 0.48 | (0.44) | 0.275 |
| day 4 × Hormone | 0.64 | (0.63) | 0.309 |  | 0.71 | (0.48) | 0.138 |
| day 5 × Hormone | 1.08 | (0.63) | 0.087 |  | 0.06 | (0.54) | 0.916 |
| day 6 × Hormone | 1.22 | (0.86) | 0.157 |  | 0.09 | (0.48) | 0.858 |
| day 7 × Hormone | 1.01 | (0.62) | 0.101 |  | 0.15 | (0.49) | 0.761 |
| day 8 × Hormone | 1.05 | (0.62) | 0.091 |  | 1.24 | (0.56) | 0.026 |
| day 9 × Hormone | 1.03 | (0.72) | 0.154 |  | 0.24 | (0.48) | 0.621 |

**^†^**Results for Model 1 reflect analyses of n = 121 hormone data points from n = 16 fathers. Results for Model 2 reflect analyses of n = 164 hormone data points from n = 19 mothers.

^‡^Hormone: a dichotomous variable indicating whether the dependent variable data point is oxytocin or testosterone.

^§^Provider: fathers’ peer ranking scores for indirect care; Dispute: fathers’ peer ranking scores for marital conflict; Direct: fathers’ peer ranking scores for direct care.

^¶^We treated age of youngest child as a continuous variable for fathers. As described in the Methods, for mothers we treated youngest child as a dichotomous variable indicating whether they had a child who was two years old or less versus a youngest child who was older than two years (comparison group).
